# Prevention of Diabetes in NOD Mice by Repeated Exposures to a Contact Allergen Inducing a Sub-Clinical Dermatitis

**DOI:** 10.1371/journal.pone.0010591

**Published:** 2010-05-11

**Authors:** Kaare Engkilde, Karsten Buschard, Axel Kornerup Hansen, Torkil Menné, Jeanne Duus Johansen

**Affiliations:** 1 National Allergy Research Centre, Department of Dermato-Allergology, Gentofte Hospital, University of Copenhagen, Gentofte, Denmark; 2 Bartholin Institute, Rigshospitalet, Copenhagen, Denmark; 3 Department of Disease Biology, Faculty of Life Sciences, University of Copenhagen, Copenhagen, Denmark; Centre de Recherche Public de la Santé (CRP-Santé), Luxembourg

## Abstract

**Background:**

Type 1 diabetes is an autoimmune disease, while allergic contact dermatitis although immune mediated, is considered an exposure driven disease that develops due to epicutanous contact with reactive low-molecular chemicals. The objective of the present study was to experimentally study the effect of contact allergens on the development of diabetes in NOD mice. As the link between contact allergy and diabetes is yet unexplained we also examined the effect of provocation with allergens on Natural Killer T (NKT) cells, since involvement of NKT cells could suggest an innate connection between the two diseases.

**Method:**

NOD mice 4 weeks of age were exposed, on the ears, to two allergens, p-phenylenediamine and 2,4-dinitrochlorobenzene respectively, to investigate the diabetes development. The mice were followed for a maximum of 32 weeks, and they were either repeatedly exposed to the allergens or only sensitized a week after arrival. The stimulation of NKT cells by the two allergens were additionally studied in C57BL/6 mice. The mice were sensitized and two weeks later provocated with the allergens. The mice were subsequently euthanized at different time points after the provocation.

**Results:**

It was found that repeated application of p-phenylenediamine reduced the incidence of diabetes compared to application with water (47% vs. 93%, P = 0.004). Moreover it was shown that in C57BL/6 mice both allergens resulted in a slight increment in the quantity of NKT cells in the liver. Application of the allergens at the same time resulted in an increased number of NKT cells in the draining auricular lymph node, and the increase appeared to be somewhat allergen specific as the accumulation was stronger for p-phenylenediamine.

**Conclusion:**

The study showed that repeated topical application on the ears with a contact allergen could prevent the development of diabetes in NOD mice. The contact allergens gave a non-visible, sub-clinical dermatitis on the application site.

The preventive effect on diabetes may be due to stimulation of peripheral NKT cells, as shown for provocation with p-phenylenediamine in the C57BL/6 mouse. This epicutanous procedure may lead to new strategies in prevention of type 1 diabetes in humans.

## Introduction

Type 1 diabetes is an autoimmune disease in which the β-cells in the pancreas are destroyed. The incidence of this disease is increasing [1], and it is therefore of interest to investigate environmental factors which could modulate the development of disease. Non-Obese Diabetic (NOD) mice are used to study type 1 diabetes in the animal setting, as female NOD mice spontaneously develop a disease equivalent to type 1 diabetes. In female NOD mice, diabetes onset occurs typically from 12 to 14 weeks of age, at which time a large number of β-cells have been destroyed. From as early as 3 to 4 weeks of age mononuclear infiltrates can be seen in the pancreas, and when they reach 25–30 week of age, around 80% of female NOD mice have diabetes [2], [3]. Factors that help promote a type 1 cytokine over a type 2 cytokine response or disturb this balance, can directly affect diabetes susceptibility in NOD mice [2].

Type 1 diabetes is considered a Th1 disease and has therefore been inversely associated with type I allergies. However, since most β-cells are already destroyed when type 1 diabetes presents, the Th1 driven pancreatic inflammation diminishes with length of disease and therefore the inverse association is probably found only in pre-diabetics and newly diagnosed patients [4]. Asthma, however, is apparently still inversely associated with type 1 diabetes and autoimmune diseases in general [5], [6].

Allergic contact dermatitis is considered a type IV delayed type hypersensitivity reaction driven by skin exposures to contact allergens. These are often present in consumer products and allergic contact dermatitis can therefore be considered an exposure driven disease. The allergens are haptens which due to their size and lipophilic nature can penetrate the skin and interact with proteins making the hapten-protein molecule immunogenic [7]. The standard method for estimating the relative potency of contact allergens is the Local Lymph Node Assay (LLNA) [8]. Recent studies using potent contact allergens have shown that CD8^+^ T cells are the effector cells of allergic contact dermatitis in mice, whereas CD4+ T cells behave as down-regulatory cells [9]–[12].

We have previously in an epidemiological study shown an inverse association between type 1 diabetes and allergic contact dermatitis [13]. On the background of this finding, the idea for the present study was conceived in which we examine whether exposure to a contact allergen can prevent the development of diabetes in NOD mice. The allergens were chosen so that they were potent as well as representing both an experimental allergen and a clinical relevant allergen 2,4-dinitrochlorobenzene (DNCB) and p-phenylenediamine (PPD) respectively.

It has been established that stimulation of Natural Killer T (NKT) cells can protect NOD mice against diabetes [3], [14]. As contact allergens stimulates NKT cells [15]–[17], we additionally explored whether the used allergens actually did influence NKT cells.

## Materials and Methods

The Danish Animal Experimental Council approved the following experiments.

### Local Lymph Node Assay (Prestudy in NOD mice)

The local lymph node assay protocol (LLNA) was performed as previously described [18]. Briefly 28 NOD/BomTac mice (Taconic, Ry, Denmark) aged 10 weeks were exposed on both ears to one of the allergens, either p-phenylenediamine (PPD) or 2,4-dinitrochlorobenzene (DNCB) (Sigma-Aldrich, Brøndby, Denmark) or with vehicle. The vehicle was acetone and olive oil 4∶1 (AOO). For the PPD groups the concentrations were 1.0, 0.25, and 0.05% (w/v), and for the DNCB groups the concentrations were 0.25, 0.05, and 0.01% (w/v). Application was done daily for three days followed by two days rest. On the sixth day the mice were injected with tritiated thymidine (TRA310, GE Healthcare, Brøndby, Denmark), and 5 hours later the mice were euthanized and the draining auricular lymph node (A-LN) removed. Using linear regression the concentration needed to induce a stimulation three times the vehicle stimulation was estimated (EC3).

### Diabetes incidence study

To study the incidence rate of diabetes in contact-allergen exposed mice, we took receipt of 104 female, 3-week-old NOD/BomTac mice (Taconic, Ry, Denmark). The mice were allocated to seven groups (Table 1). These groups were further allocated to exposure with PPD, DNCB, vehicle (AOO) or water (one group). All groups received 3 topical exposures a week after being delivered, analogous to the first three days of the LLNA.

**Table 1:** Concentrations and groups in the diabetes incidence study.

Exposure substance	PPD	PPD	DNCB	DNCB	AOO	AOO	Water
Groups	Induction only	Repeated exposure	Induction only	Repeated exposure	“Induction” only	Repeated exposure	Repeated exposure
Conc. 4^th^ week	0.1%(w/v)	0.1%(w/v)	0.1%(w/v)	0.1%(w/v)	Pure solvent	Pure solvent	Pure solvent
Conc. Rest of the weeks	No exposure	0.01%(w/v)	No exposure	0.01%(w/v)	No exposure	Pure solvent	Pure solvent

Four groups were further exposed repeatedly every two weeks with a single application until a maximum of 32 weeks of life. These groups consisted of one group from each of the PPD, DNCB and AOO exposure groups and the water exposure group (Table 1).

The first exposure, on the fourth week of life, was epicutaneous application of 25 µL of a concentration of 0.1%(w/v) of the allergens (PPD or DNCB) and pure solvent for the other exposure groups (AOO and water). For the repeated exposures (four groups) the amount remained at 25 µL, whereas the concentration of allergen was reduced to 0.01%(w/v). The vehicle (AOO) and water repeated exposure groups received only AOO or water, respectively. The dilutions were freshly made immediately before application.

**Figure 1:**
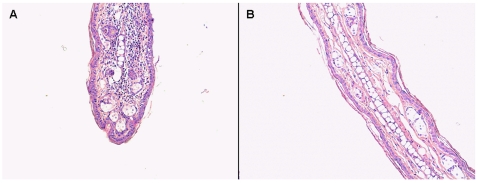
Mononuclear infiltrations in the ears after exposure to contact allergen. The figure shows hematoxylin and eosin stained ears from a mouse in the group of mice repeatedly exposed to PPD and provoked with a higher dose at the end of the study (A), and from a mouse from the group repeatedly exposed to DNCB (B). The H&E stained DNCB ear is representative of the repeatedly exposed mice. The H&E stained PPD ear shows an increase in infiltration which rules out that they were tolerized to the allergen and the reduced incidence could be due to bystander suppression.

The experiment ended when the mice were 32-weeks old, at which time the mice in the repeatedly exposed groups had received 14 applications of the repeated dose. At 12 weeks of age, weekly measurements of tail blood glucose commenced. If the glucose level was equal to or above 14 mmol/L on two consecutive days, the mice were considered to be diabetic and were euthanized. At the end of the study the mice still alive in the PPD repeatedly exposed group were provoked with 25 µL, 0.1% (w/v) PPD, and euthanized two days later. A histological examination was made to assess cellular infiltration of the ears (Fig. 1). All samples were fixed in formalin and stained in hematoxylin and eosin. The rest of the mice still alive at the end of the study, were euthanized and their ears were also examined for cellular infiltration. Additionally we did a cytokine analysis of blood serum drawn by cardiac puncture at euthanization. The analysis was done by use of the flow metric Luminex xMAP technology using beads for the following cytokines TNF-alpha, IL-1beta, IFN-gamma, IL-4, IL-5, IL-6, IL-10, IL-12 (p40), IL-12 (p70), IL-13, IL-17, MIP-1, TGF-β were from Panomics (Panomics, Inc., Redwood City, CA, USA). TGF-β was run on the same plates as the other targets. The staining was done according to the manufactures guideline.

The cumulative diabetes incidence was evaluated by the Kaplan-Meier estimation, and the significance was estimated by the log-rank test. The analysis was done with SPSS version 15.0 (SPSS, Chicago, IL, USA)

### NKT cell study

We took receipt of 10-week-old female C57BL/6JBomTac mice (Taconic, Ry, Denmark). One week after arrival 10 mice were sensitized with 0.5% (w/v) DNCB in AOO and 10 mice with PPD 0.5% (w/v). Two weeks after sensitization the mice were separated into ten groups of two mice–accordingly there were five groups treated with either allergen. In eight groups the respective allergen in a concentration of 0.5% (w/v) was re-applied to both ears. At the following time points after re-application (18, 24, 48, and 72 hours) a group of mice was anaesthetized, fixated and vena portae was ligated. Vena cava was cut and the mice bled. Subsequently the liver was perfused with PBS containing heparin until pale; afterwards the liver was removed along with the draining auricular lymph node (A-LN). The groups which did not receive a reapplication, the control groups, were euthanized at 48 hour.

**Figure 2:**
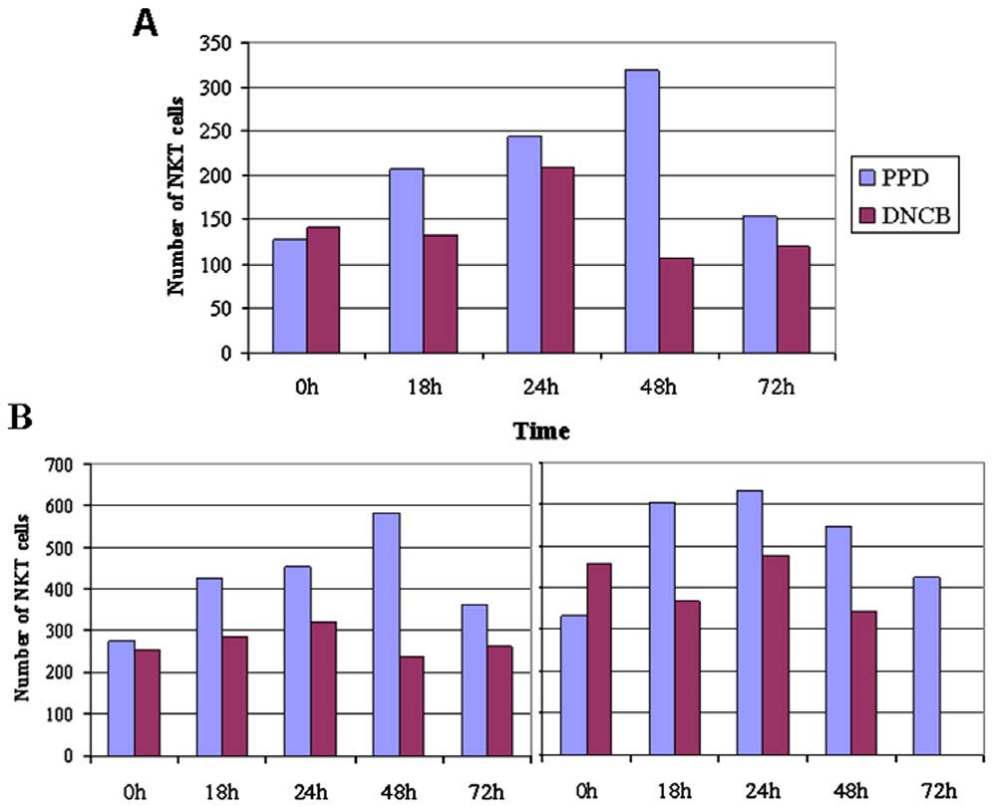
Topical exposure stimulates NKT cells in A-LN's. The figures show the number of NKT cells in the A-LN. The A-LN cells were gated to exclude nonviable cells and sample data were collected on 300,000 cells. It can be seen that both allergens stimulates NKT cells in the A-LN though PPD appear to be a stronger or longer stimulator. The increase in cells are followed by a steep drop that may be due to down regulation of the T cell receptor which have been reported previously [35]. Panel A and B shows different experiments. A Mann-Whitney U test was conducted on the experiment in panel A while panel B is included to show other experiments. The Mann-Whitney U tests performed on the experiment in panel A was performed on both the quantity of NKT in relation to the control groups (0 h) and on the number of NKT cells. For the quantity of NKT cells the Mann-Whitney U test was significant (z = −2.095, P<0.05) while the result for the number of NKT cells was insignificant (z = −1.567, P>0.05).

The livers and A-LN's were strained (70 µm; BD Biosciences, Brøndby, Denmark), to gain a single cell suspensions, and the liver lymphocytes were isolated using Lympholyte-M (CEDARLANE Laboratories, Burlington, Canada). Subsequently the cell suspensions were stained with APC-conjugated CD1d tetramers (ProImmune, Oxford Science Park, UK) loaded with α-galactosylceramide (Alexis Biochemicals/Axxora, San Diego, USA) according to the manufacture guidelines. Thereupon the following cellular receptors were stained; FITC conjugated anti-CD3 (BD Biosciences, Brøndby, Denmark), PE-Cy5.5 conjugated anti-TCR-β, PE-Cy7 conjugated anti-NK1.1 (eBioscience, San Diego, USA) and APC-Cy7 conjugated CD19 (BioLegend, San Diego, USA). Afterwards the cells were analyzed on a FACSCanto II (BD Biosciences, Brøndby, Denmark). The CD19 positive cells were gated out, and TCR-β positive cells were selected. Subsequently cells in the quadrant containing CD3 positive and CD1d-tetramer-aGC-positive were designated as CD1d restricted NKT cells. The number of cells in the A-LN's in response to the different exposures can be seen in figure 2. The quantity of NKT cells were additionally determined as the proportion between NKT cells at the different time points and the control groups. This was done as there are very few NKT cells in the A-LN, and there was a small difference in the number of NKT cells in the control groups (0 h) (Fig. 2A). Difference between the two exposures was compared using the Mann-Whitney U test.

## Results

By doing a linear regression on the results from the Local Lymph Node Assay the concentration needed to induce a stimulation three times the vehicle stimulation was estimated (EC3). The value were 0.169%(w/v) and 0.037%(w/v) respectively for PPD and DNCB. These values are close to the EC3 values previously reported for the CBA mouse [19], [20], which is recommended mouse strain in this protocol. Thus, it is possible to induce, an easily manageable dermatitis using the two allergens. On the basis of the estimated EC3 values we decided to use a concentration of 0.1%(w/v) for sensitization and 0.01%(w/v) for repeated treatment for both allergens.

**Figure 3:**
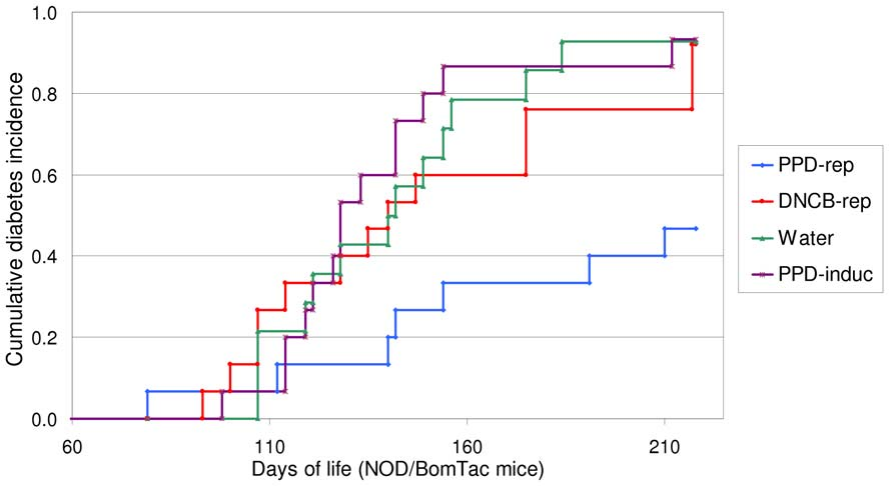
Repeated application of PPD inhibits the development of diabetes in NOD mice. The figure shows a Kaplan-Meier curve of the diabetes incidence for four groups of mice. DNCB-rep, PPD-rep and Water, refers to the groups of mice that were repeatedly exposed to DNCB, PPD or water every other week. PPD-induc refers to the group of mice that was only exposed to PPD in the fourth week of life. The NOD mice exposed to PPD repeatedly, displayed a cumulative diabetes incidence of 47%, in contrast to 93% of the water treated group (P = 0.004). The log-rank test for all the shown curves gives a significance of P = 0.008.

The diabetes incidence study revealed that NOD mice repeatedly exposed to PPD, displayed a cumulative diabetes incidence of 47%, in contrast to 93% of the water treated group (P = 0.004). The group repeatedly exposed to DNCB had an incidence of 92%, and the group which only received the PPD sensitizing regime 93% (Fig. 3). The three other groups developed diabetes by a rate similarly with a cumulative incidence from 80–86% (data not shown).

The cytokine analysis did not reveal any difference between the exposure groups, and there were no elevated cytokines that could suggest a systemic response.

Dermatitis was not noticeable by visual inspection, however sensitization in the group repeatedly exposed to PPD, was confirmed by a typical lymphocytic infiltration in the ears following PPD provocation, and a more moderate infiltrate was also noticeable in the group repeatedly exposed to DNCB (Fig. 1).

The NKT study demonstrated that both allergens influenced the quantity of NKT cells in an analogous manner in the liver (data not shown), while PPD appear to influence the amount of NKT cells more strongly than DNCB in the A-LN's. A Mann-Whitney U test was conducted to evaluate the difference between the A-LN's. The result of the test on the quantity was significant, z = −2.095, P<0.05, while the result on the number of NKT cells was insignificant, z = −1.567, P>0.05. The quantity of NKT was calculated by dividing with the number of cells in the control groups (0 h).

## Discussion

Here we describe the intriguing finding that inducing a minor non-visible dermatitis on a small area, by repeated applications of PPD, prevents the diabetes development in NOD mice. The repeated application of allergen gave a sub-clinical dermatitis. This could not be explained by an abnormal response in NOD mice to the used allergens, as the NOD mice reacted similar to the standard mouse strain used for LLNA. Moreover we found that both allergens modulated the number of NKT cells in the liver, which is in accordance with previous published data [16].

It is indicated that repeated application of a contact allergen can give a Th2 cytokine milieu [21], which may be a mechanism for the protective effect of repeated application of PPD. The lack of effect of DNCB is not clear, though it may be related to the absence of a peripheral NKT cell response compared to that of PPD. The discrepancy in the allergens ability in hindering diabetes may be due to difference in potency as there is a factor four differences between their potency, however there were no difference in ear infiltration. Furthermore a cytokine analysis of cardiac blood drawn at euthanization did not show any differences between the exposure groups, and could therefore not reveal the presences of a systemic inflammation. As the mice are sensitized at a very young age, it could be that the more potent allergen (DNCB) has somewhat tolerized the mice, and that the allergic response therefore is not strong enough to inhibit the diabetes progression. However the ear infiltration in the groups were comparable, which do not support this hypothesis, and high doses of contact allergens applied epicutanously have not previously been reported to tolerize mice. This could be due to the so-called danger signal, the irritant effect of contact allergens [22] which can causes toxic effects if the amount used is to large. High-dose tolerance have therefore be achieved though intravenous injection of contact allergens [23], [24]. Alternatively the PPD groups could have been subjected to low zone tolerance [25], however the PPD group that was treated repeatedly was provocated at the end of the incidence study and the histology of these mice ears showed that the mice were sensitized (Fig 1A). The lack of effect of DNCB hints to other experiments with lower doses of DNCB. PPD could however also be a special contact allergen, as it can induce immediate hypersensitivity [26], [27], and there might additionally be a differences in the duration of time at which the allergens are bound in the skin. PPD has been shown to have a long persistency in the skin [28]. Moreover there could be a difference in the time span of the cytokine response, as it has previously been shown in THP-1 cells that the release of IL-8 decreases much faster in cells treated with DNCB compared to treatment with PPD [29].

The peripheral stimulation of NKT cells by contact allergens is in agreement with a recent human study [17]. The mechanism for the protective effect of allergic contact dermatitis on diabetes is unknown, but activation of NKT cells could be an explanation, since up-regulation of NKT cells have been shown to protect NOD mice against diabetes [3], [14], and PPD gave a more pronounced NKT cell response in the A-LN (Fig. 2). The difference between PPD and DNCB in the peripheral activation of NKT cells might then explain the selective protective effect of PPD. For the study on NKT cells we wished to study the effect on mature 14-weeks-old mice. Therefore the C57BL/6 mouse was chosen instead of the NOD mice, as this would have required insulin treatment which potentially could influence the results. NOD mice were furthermore not chosen to study NKT cells, as NOD mice have systemic deficiency of NKT cells [30], and additional are the number of NKT cells in periphery somewhat reduced [31], hindering efficient evaluation of the effect of contact allergens on NKT cells.

This study investigates the inhibitory effect of contact allergens on diabetes development. The repeated epicutanous application of the contact allergens gave a local harmless subclinical dermatitis. The application involved only the outside of the ears, which is in contrast to the systemic and immunological unspecific treatments with anti-CD3 [32] and complete Freund's adjuvant [33] or the systemic treatment with BCG vaccination [34].

In conclusion, we show that controlled repeated epicutanous exposures to a contact allergen prevent development of diabetes in the NOD mouse. The mechanism may be a skewing of the immune system towards Th2 cells which has been described in studies where the allergen has been reapplied several times and peripheral up-regulation of NKT cells may additionally play a role in the preventive effect. This observation may lead to a new understanding of the integrated immune function and might lead to a new strategy for prevention of type 1 diabetes.
